# Predictors of Urinary Abnormalities in Children Hospitalised for Their First Urinary Tract Infection

**DOI:** 10.3390/children11010055

**Published:** 2023-12-30

**Authors:** Danilo Buonsenso, Giorgio Sodero, Anna Camporesi, Ugo Maria Pierucci, Francesca Raffaelli, Francesco Proli, Piero Valentini, Claudia Rendeli

**Affiliations:** 1 Department of Woman and Child Health and Public Health, Fondazione Policlinico Universitario A. Gemelli IRCCS, 00168 Rome, Italy; francesco.proli@guest.policlinicogemelli.it (F.P.); piero.valentini@policlinicogemelli.it (P.V.); claudia.rendeli@unicatt.it (C.R.); 2 Centro di Salute Globale, Università Cattolica del Sacro Cuore, 00168 Roma, Italy; 3 Institute of Pediatrics, Università Cattolica del Sacro Cuore, 00168 Rome, Italy; giorgio.sodero@icatt.it; 4 Department of Pediatric Anesthesia and Intensive Care, Buzzi Children’s Hospital, 20154 Milan, Italy; anna.camporesi@asst-fbf-sacco.it; 5 Department of Pediatric Surgery, Buzzi Children’s Hospital, 20154 Milan, Italy; ugo.pierucci@asst-fbf-sacco.it; 6 Dipartimento di Scienze di Laboratorio e Infettivologiche, Fondazione Policlinico Universitario A. Gemelli IRCCS, 00168 Rome, Italy; francesca.raffaelli@policlinicogemelli.it

**Keywords:** urinary tract infections, children, urinary abnormalities

## Abstract

We aimed to investigate if children with their first UTI and a concomitant positive blood culture have a higher risk of abnormalities. We performed a retrospective study of children younger than 18 years of age with their first UTI. Multivariate logistic regression and receiver operating characteristic (ROC) curves were used to evaluate if positive blood cultures are associated with urinary abnormalities. After the screening process, we considered the enrolled 161 children with UTIs. The median age was three months, and 83 were females (43.2%). In multivariate analysis, age (*p* = 0.001, 95% CI 1.005–1.020), the presence of Pseudomonas aeruginosa or unusual germs in urine cultures (*p* = 0.002, 95% CI 2.18–30.36) and the positivity of blood cultures (*p* = 0.001, 95% CI 2.23–18.98) were significantly associated with urinary abnormalities. A model based on these parameters has an AUC of 0.7168 to predict urinary malformations (*p* = 0.0315). Conclusions include how greater age, a positive blood culture and the presence of Pseudomonas aeruginosa or unusual germs in urine culture in children hospitalised for their first episode of a UTI are factors associated with a significantly higher risk of urinary abnormalities. These data can guide the implementation of more personalized strategies to screen for urinary abnormalities that may be included in future guidelines.

## 1. Introduction

Urinary tract infections (UTIs) are one of the most frequent infections in children [1]. Mild cases can be managed at home, while severe forms often require hospitalisation and intravenous antibiotic therapy to prevent complications [2]. The clinical presentation of UTIs includes dysuria, increased frequency and urgency of voiding, pyuria with fever, nausea, vomiting, irritability, and refusal to eat [3]. Although the localisation is associated with several clinical symptoms, it is difficult to differentiate UTI localisation (upper versus lower urinary tract) on the basis of clinical symptoms and signs, especially in neonates and young children [4]. Untreated or undiagnosed infections can evolve into urinary sepsis, a condition that puts the child’s life at risk and requires hospitalization and intravenous antibiotic therapy [2].

Inflammation markers, such as C-reactive proteins (CRPs), often increase with neutrophilic leukocytosis and alterations upon urinalysis alongside other indirect signs of infection such as leukocyte esterase, nitrites or haemoglobin [5]. Procalcitonin (PCT) can also be used to identify the most seriously ill patients that require hospitalisation and it also has good sensitivity and specificity in predicting the contextual bloodstream infection and other late onset complications [6]. Increased creatinine levels upon presentation also can predict the risk of renal parenchymal involvement and bacteriemia [7], identifying patients who require broad-spectrum antibiotic therapy and prolonged hospitalization.

UTIs can affect children with normal anatomy or, in some cases, can be the first symptom of a urinary tract abnormality [8] which requires an adequate urological follow-up. The abnormality can be congenital (urinary malformations) or acquired (such as, for example, most forms of kidney scarring that can affect patients with pyelonephritis). Ultrasonography is generally the first diagnostic tool when urinary abnormalities are suspected and is performed days to weeks after the acute episode, while cystourethrography is recommended in case of ultrasound abnormalities or in recurrent infections to exclude vesicoureteral reflux (VUR) [9].

The role of screening radiological examinations following the first episode of a UTI is controversial. There are several guidelines and recommendations from various scientific societies with sometimes conflicting indications. For example the American Academy for Pediatrics (AAP) [10] recommends renal and bladder ultrasonography for children between two months and two years of age after a first time febrile UTI, while National Institute for Health and Care Excellence (NICE) guidelines [11] recommend it only in children younger than six months and with a recurrent UTI or with atypical features.

European Association of Urology (EAU) guidelines recommend an early ultrasound screening within 24 h to exclude any possible urinary tract obstructions or other genitourinary anomalies which are diagnosed in 15% of cases. Of these cases, about 2% require immediate treatment. In the presence of an abnormality detected using an ultrasound in patients under two years old, all guidelines recommend a voiding cystourethrography. In most cases, the decision is individualized depending on the patients’ characteristics and on their clinical response to antibiotic therapy.

It is possible that many cases of urinary abnormalities are not identified after the first episode of a UTI but are detected later in the event of a recurrence, exposing the child to a new infection with consequent antibiotic treatment, hospitalization and possible complications. On the other hand, screening all first episodes of UTIs is not cost effective, although it would allow effective prevention using antibiotic prophylaxis or corrective surgery [12]. Furthermore, the relationship between blood infections and the presence of underlying urinary abnormalities is controversial. In fact, complicated UTIs can at the same time be more frequent in case of alternations of the anatomy of the urinary tract (as in the case of patients with dilations from vesicoureteral reflux), but themselves can also cause kidney alterations following the inflammation process (renal scars, abscesses and long-term complications such as hypertension or kidney failure) [6].

In this study, we hypothesized that children having bacteremia during their first episode of a UTI may have an increased risk of urinary abnormalities. This is an important hypothesis to test because, if confirmed, this may guide the clinician in performing an ultrasound assessment in the case of an initial episode of a UTI.

## 2. Materials and Methods

This is a retrospective study of children younger than 18 years who were hospitalised in the paediatric department with a first known episode of a UTI from March 2017 to April 2023. In our centre, reasons for admission for a UTI are an inability to retain fluids, dehydration requiring intravenous fluids, and being ill- or septic-appearing. All patients were followed up, according to our routine practice, for at least three months after admission into our outpatient paediatric urologic service to evaluate the reappearance of signs or symptoms compatible with new infections and to perform, when necessary, radiological tests (urinary tract ultrasound, cystourethrography, renal scintigraphy). Therefore, we have been able to detect urinary abnormalities also in those children that were not tested with imaging before the acute episode.

We used an excel database to collect our data. We retrieved the following information from electronic medical records for each patient with regard to age, results of blood tests performed upon admission (C-reactive protein, procalcitonin), results of microbiological tests (blood culture, urine culture) and results of radiological screening performed in the three months following a UTI episode.

### 2.1. Aim

The aim of this study is to evaluate if, in children with their first episode of a UTI, a positive blood culture is associated with a higher risk of urinary abnormalities.

### 2.2. Inclusion Criteria

Our target population was composed of children hospitalised for their first episode of a UTI defined as follows:

Positive urinalysis (presence of nitrite and/or leukocyte esterase) in one urine sample collected through bladder catheterization or clean-voided urine and positive urine cultures for a single bacterium with (>104 CFU/mL) (or, in cases of more than one germ being isolated with at least one of them having a charge of >104 CFU/mL and where contamination was excluded by paediatricians on the basis of the bacterial load and the type of pathogen).

Positive urine cultures for a single bacterium (>104 CFU/mL), even in the case of normal urinalysis, if the child had compatible signs and symptoms and blood laboratory findings and no other alternative diagnoses were obtained, and the treating clinicians diagnosed the child and treated them for a UTI and the child was discharged with only the diagnosis of a UTI.

In our study, we did not consider patients with a positive urine culture from urinary bag specimens due to the possible risk of contamination. We included only children that had a complete follow-up for at least three months in the Paediatric Urologic Disorders Unit. According to our local guidelines and hospital protocols, all patients underwent a urinary tract ultrasound at least seven days after UTI hospitalization, while voiding cystourethrography and renal scintigraphy were performed when deemed necessary by the paediatric urologist.

In our study we considered the following urinary abnormalities, diagnosed by ultrasound and/or voiding cystourethrography, which were classified as “urinary abnormalities” in our dataset:

Mono or bilateral pelvicalyceal dilatation (2° Society of Fetal Urology grade hydronephrosis).
Double renal pelvis.
Ureteral dilatation (ureterocele, pyeloureteral junction syndrome).
Bladder abnormalities (ureterocele, diverticulum).
Vescico-ureteral reflux.
Unilateral or bilateral renal abnormalities (hypoplasia, multicystic kidney).

### 2.3. Ethic Committee

The study was reviewed and approved by the Human Research Ethics Committee of the Fondazione Policlinico Universitario A. Gemelli IRCCS of Rome, Italy, as part of a larger study that evaluated clinical and laboratory characteristics of children hospitalised for a UTI (prot 0010122/22, ID 4808). The study was conducted in accordance with the Declaration of Helsinki and its subsequent amendments. The parents of all patients were informed about the purposes of the data collection and signed to give their informed consent. No personal or identifiable data were collected during the conduct of this study.

### 2.4. Statistical Analysis

Categorical variables were described as frequencies and percentages and continuous variables expressed as a mean (SD) or median (IQR) as appropriate. We used chi-square tests to analyse categorical variables and Student’s t test or the Wilcoxon rank-sum test, as appropriate, for continuous variables. Statistical significance was designated as *p* value ˂ 0.05 (2-sided).

The occurrence of genitourinary abnormalities was considered as dependent variables in a logistic multivariate regression model. The model was tested on the following potentially predictive variables of age (months), female sex, type of germ in the urine culture, positive blood culture, occurrence of the same germ in both blood and urine, C-reactive protein (mg/dL) values, procalcitonin values.

Receiver operating characteristic (ROC) curves were used to test the ability to predict the presence of genito-urinary abnormalities in two models, differing for the presence of positive blood cultures. Areas under the ROC curves were compared using DeLong’s method. Calibration was tested using the Hosmer–Lemeshow goodness-of-fit test. All analyses were carried out using Stata v18.0 (StataCorp LP, College Station, TX, USA).

## 3. Results

**Figure 1:**
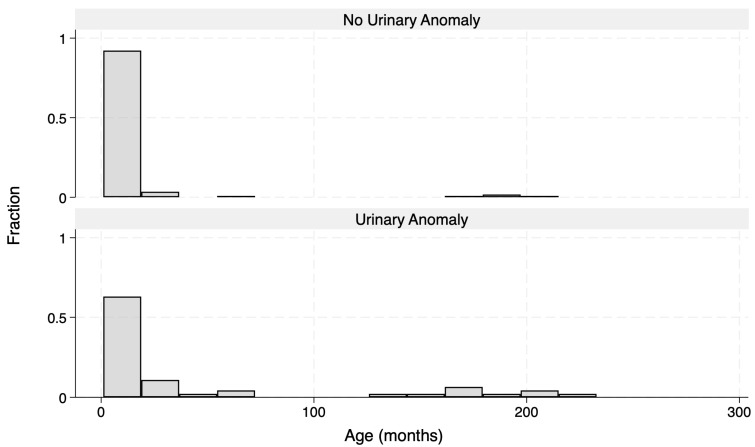
Correlation between age and urinary anomaly frequency.

A total of 161 patients were enrolled into the study. Demographic and microbiological data of the cohort are reported in Table 1. Age distribution was different between those who were then diagnosed with a urinary anomaly and those who were not (Figure 1).

Forty-six patients had the final diagnosis of a urinary tract abnormality (21/46 pelvicaliceal dilatations, 4/46 renal double pelvis, 6/46 ureteral dilatations, 9/46 VUR, 6 bladder abnormalities). Of all patients, 19 (12%) also presented a positive blood culture, 18/19 of them had the diagnosis of urinary tract abnormalities and 8 presented the same germ in urine and blood. During follow-up periods, about 21 had pelvic dilations, 17 required a surgical follow-up and subsequent pyeloplasty (which was successful in all cases), while the remaining four were only periodically followed up using a renal ultrasound. Of the four cases of a renal double pelvis, three were followed up with an ultrasound while only one underwent corrective surgery due to the presence of VUR. All six ureteral dilatations were associated with vescico-ureteric junction obstruction and were corrected surgically. Of the nine cases of VUR, four were followed up over time (low grade) and resolved spontaneously, while five (high grade) required continuous antibiotic prophylaxis and subsequent surgery. None of the six bladder abnormalities required surgical correction. All patients are currently being followed up at our centre.

CRP values did not differ between those with a final diagnosis of urinary abnormalities and those without and between those with a positivity on blood culture and those without. PCT, on the other hand, was significantly different between those groups and was also significantly different between those who had a positive blood culture and a final diagnosis of urinary abnormalities and those who did not present either of these characteristics (Table 2).

Logistic regression was performed following the outcome of a “diagnosis of urinary abnormalities” using a step-down method and considering the different bacteria grown in the urine cultures. From urine culture growth, the presence of *Pseudomonas aeurignosa* or the presence of unusual bacteria, such as *E. fergusonii*, or fungi, such as *Candida albicans*, resulted in being significative risk factors, while the presence of *E. coli* or Klebsiella was not. We tested multivariate models including one only for Pseudomonas, which had a greater incidence in our cohort, and one for the presence of either Pseudomonas or unusual germs.

**Figure 2:**
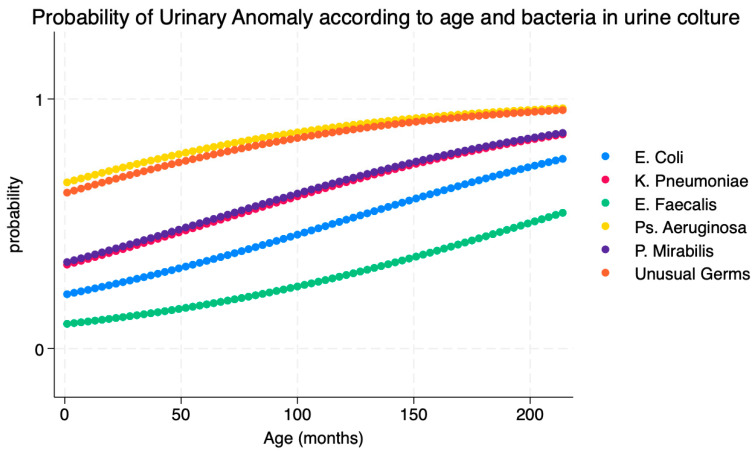
Relationship between age and different bacteria retrieved in urine cultures in predicting a urinary abnormality.

**Figure 3:**
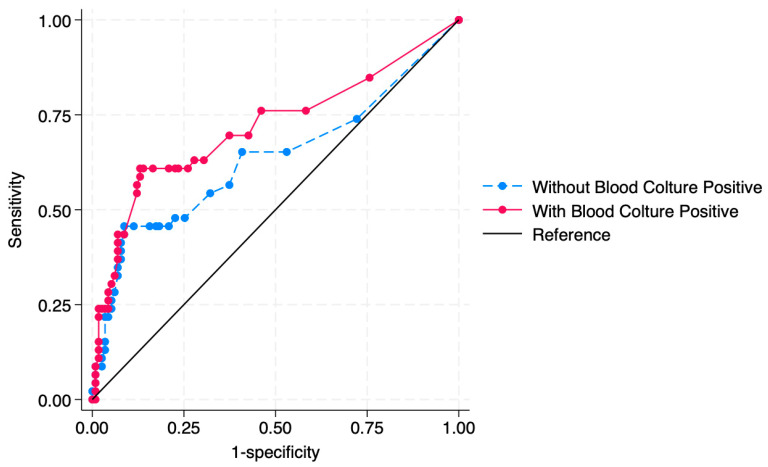
AUC of the model used to predict urinary abnormalities. Inclusion of positivity on blood culture increases the AUC from 0.6349 to 0.7168.

The best model included age, the presence of Pseudomonas aeruginosa or unusual germs in urine culture and positivity of blood culture (Pseudo R2 = 0.1679) as risk factors. Table 3 describes the results of the multivariate analysis. Greater age increases the risk of presenting urinary abnormalities by 1% for every month of growth; a urine culture positive for *P. aeruginosa* or unusual germs and blood culture positivity are associated with a risk of urinary abnormalities. Figure 2 describes the probability of diagnosing a urinary abnormality in the patient according to the different bacteria retrieved in a urine culture. Figure 3 describes the relationship between the age and the different bacteria retrieved in urine cultures in predicting the outcome of “diagnosis of urinary abnormality”. Table 4 describes the predicted probability of a urinary anomaly according to the bacteria encountered in the urine culture while holding other covariants as constant.

In these models the presence of a positive blood culture improved the AUC from 0.6349 to 0.7168 (*p* = 0.0315).

## 4. Discussion

To our knowledge, our study is one of the first to analyse the possible correlation between age, urine and blood cultures with urinary abnormalities in a cohort of hospitalised paediatric patients suffering from their first episode of a UTI. We found that greater age, presence of Pseudomonas aeruginosa or unusual germs in urine culture and positivity of blood culture were significantly associated with urinary abnormalities. We also found that there may be a specific risk of urinary malformations according to the type of infection and the age of the initial UTI. This is an ever-evolving topic, with recommendations being constantly updated based on recent evidence. About 7.0% of febrile and 7.8% of children with urinary symptoms have a UTI [13]. Therefore, our findings may inform the personalisation of future protocols for the identification of children hospitalised for their first UTI as being at a higher risk of urinary malformation.

According to the Italian guidelines for the management of UTIs [14], the use of a urine collection bag is recommended only for a urine dipstick as the probability of contamination is high, while catheterization is currently the most sensitive and specific method, especially in young infants and newborns [15]. Therapeutic management often requires an oral antibiotic treatment based on local sensitivity patterns for common uropathogens, with a culture taken prior to initiation and a change in antibiotic enacted if the culture results indicate the organism is resistant to the initial antibiotic chosen [16]. Meanwhile, intravenous treatment is indicated for severe UTIs with a higher risk of complications [2]. Today, new personalised therapeutic regimens are also possible with different and/or shorter durations customised to the patient’s characteristics (such as an early response to antibiotic therapy) and on the early reduction of inflammation indices [17].

Bloodstream infections still remain an important cause of mortality and morbidity during paediatric age. Clinical presentation is variable and, in fact, could be asymptomatic during the initial stages and could lead to multiorgan failure and septic shock [18], of which the ones associated with UTIs, commonly called urosepsis, may be present alongside urinary symptoms or a decline in the child’s general condition [4]. The relationship between urosepsis and urinary anomalies is bilateral as complicated UTIs are more frequently associated with malformations of the urinary tract and more often cause subsequent anomalies such as renal scarring [6].

In our study, we hypothesized that the presence of a positive blood culture is associated with a higher risk of having concomitant urinary abnormalities (congenital or acquired). Our data confirm the initial hypothesis, since the multivariate analysis age, the presence of Pseudomonas aeruginosa or unusual germs in urine culture and the positivity of blood cultures were significantly associated with urinary abnormalities. Therefore, these patients are worthy of an accurate radiological follow-up in order to prevent future episodes of UTIs and complications through implementing prevention strategies, such as antibiotic prophylaxis or corrective surgery, when necessary. Interestingly, we have also been able to describe an association between infections and specific pathogens, age and the probability of urinary abnormalities, finding that these associations are relative and not absolute. If these findings are confirmed in larger multicenter studies, a more personalized model could predict the risk of having urinary malformations for each child in a specific age group and for each specific infection and therefore predict the cost-effectiveness of performing screening tests. To our knowledge, such a finding is new for the available literature.

Regarding the use of radiological examinations after the UTI episode, a recent publication [19] has highlighted that the application of different guidelines is associated with a missed diagnosis of several concomitant anomalies and complications of the infection. The authors performed a retrospective analysis of 43 children hospitalised for a UTI with a complete radiological follow-up (ultrasound, cystourethrography and renal scintigraphy), highlighting that the retrospective application of NICE [11] guidelines resulted in a reduction of 9% in ultrasounds, 51% in cystourethrographies and 53% in renal scintigraphy tests alongside a significant missed number of cases of VUR, hydronephrosis and renal scarring. Meanwhile, the application of AAP guidelines [10] did not reduce the number of ultrasound examinations and the consequent diagnosis of hydronephrosis. Despite the authors’ results, this is a retrospective study and most international guidelines state that prenatal screening ultrasounds during the third trimester of pregnancy are able to diagnose most major urinary abnormalities, while minor anomalies and cases of unidentified low-grade VUR would not justify the application of voiding cystourethrography due to the invasiveness of the procedure.

In our centre, a urinary tract ultrasound is performed about seven or more days after UTI hospitalization while voiding cystourethrography and renal scintigraphy were performed when deemed necessary by the paediatric urologist. Our recommendations are in line with local guidelines [14] that recommend an ultrasound evaluation for all children 2–4 weeks after their first febrile UTI to identify renal and urinary tract abnormalities. Screening for VUR is generally performed in the presence of ultrasound abnormalities, except in the case of isolated dilations of the renal pelvis which can be monitored over time.

Screening all patients with a previous diagnosis of a UTI with an ultrasound examination allows an early identification of urinary anomalies but is not cost effective. It is therefore necessary to find new selection criteria for children who need early radiological screenings; the presence of a positive blood culture could be one of the markers to take into consideration.

In a study conducted on 43 children aged between one month and 14 years, the increase in D-Dimer and procalcitonin was related to upper urinary tract involvement (PCT ≥ 0.255 ng/mL sensitivity 50% specificity 80% PPV 89.5% PNV 33.3%) [20] and the possible presence of renal parenchymal involvement; therefore, these indices could be used to classify patients with early imaging needs.

Our study also showed a correlation between urinary abnormalities and the presence of a positive blood culture in addition to age and non-e.coli bacteria in urine cultures. In a study of 343 patients with urinary tract infections, of whom 249 were found to have a positive blood crop, a different prevalence of bacteremia was observed among patients less than two months of age compared with those of the age of and up to three years (22.7% vs. 3%) [21]. These results confirm the increased severity of neonatal infections, which are more frequently complicated through sepsis, but there are no data on the prevalence of urinary abnormalities.

In patients with complicated bacteremia infections, urinary abnormalities are much more frequent than uncomplicated infections. For example, Seo Hee Yoon et al. Twenty-two [22] showed that those with a bacteremic UTI had a significantly higher prevalence of VUR compared to those with a non-bacteremic UTI (59.3% vs. 30.6%; *p* = 0.003); therefore, the early use of imaging and cystourethrography in these patients may be justified.

It is clear that the early application of screening methods leads to early diagnosis and treatment; however, it is controversial how much this changes the prognosis of these patients. In fact, notwithstanding increasingly accurate diagnostic methods, a meta-analysis has shown rates of children with reflux nephropathy progressing to end-stage renal disease are not declining despite current preventive and therapeutic strategies [23]. Antibiotic oral prophylaxis could prevent numerous UTI reinfections in children with urinary abnormalities but could also increase the antibiotic resistance of pathogenic bacteria [24]. A study involving 607 children with VUR [25], diagnosed either after the first or second symptomatic urinary tract infection, highlighted that trimethoprim–sulfamethoxazole administered with good adherence reduced the risk of recurrences by 50% (hazard ratio, 0.50; 95% CI, 0.34 to 0.74) without a reduction in the occurrence of renal scarring, although cases of re-infections from *Escherichia coli* were more frequently resistant to trimethoprim–sulfamethoxazole in the prophylaxis group compared with the placebo group.

A recent randomized controlled trial [26] conducted on 292 infants with vesicoureteral reflux (diagnosed before the first episode of UTI) showed a significant benefit of continuous antibiotic prophylaxis in preventing urinary infection (hazard ratio, 0.55; 95% confidence interval [CI], 0.35 to 0.86; *p* = 0.008). However, the incidence of renal scarring was not different between the two groups. Furthermore, a collateral increase in urinary infections caused by uncommon germs or from bacteria resistant to classic antibiotic therapies was highlighted. In clinical practice, considering the risk of patients with high-grade VUR, antibiotic prophylaxis is generally recommendable despite the possible risk of these adverse events.

A possible alternative to antibiotic prophylaxis is the administration of probiotics after the first UTI episode [27] which, although there is conflicting evidence [27], appear to be effective in children with normal urinary tract anatomy. Meanwhile, there are no data on the efficacy in patients with abnormalities or with a previous bloodstream infection.

Our study has limitations to address. Despite the relatively large number of patients enrolled, this is a single-centre retrospective study; therefore, the results of our statistical analysis may not be generalizable to the entire paediatric population. Moreover, we only analysed hospitalised patients which represent a more severe spectrum of UTIs. Thus, our results may not be translated into children managed in outpatient settings. Furthermore, we did not distinguish acquired urinary abnormalities from other congenital malformations due to the lack of information about prenatal ultrasound exams. It is therefore possible that a subgroup analysis could lead to different results. Lastly, a relatively small proportion of patients had positive blood cultures (about 1/4), or *P. aeroginosas* or other uncommon pathogens (about 1/4) and urinary malformations; therefore, studies on larger number of children with UTIs and positive blood cultures or uncommon bacteria are needed to confirm our findings.

## 5. Conclusions

In our sample of children hospitalised for their first episodes of UTIs, a positive blood culture and the presence of *Pseudomonas aeruginosa* or unusual germs in urine culture were factors associated with a significantly higher risk of urinary abnormalities. These data, if confirmed through a prospective multicenter study, may lead to the modification of guidelines for the timing of imaging diagnostics in a subgroup of children with their first episode of UTIs who are at a higher risk of urinary alterations. This would be in order to identify urinary malformations early and to establish an adequate follow-up.

## Figures and Tables

**Table 1:** Demographic and microbiologic data of the cohort according to final outcome. Data are presented as a mean (SD) or median (IQR) for continuous measures as appropriate and n (%) for categorical measures. Others *: *S. agalactiae* (1); *Candida albicans* (1); *K. aerogenes* (1); *E. fergusonii* (1).

	Total	No Urinary Abnormalities	Urinary Abnormalities	*p*-Value
	N = 161	N = 115	N = 46	
Female sex	68 (42%)	52 (45%)	16 (35%)	0.23
Age (months)	3 (1–8)	3 (1–6)	4 (1–48)	0.061
Urine culture Bacteria				0.23
*E. coli*	124 (77%)	90 (78%)	34 (74%)	
*K. oxytoca*	3 (2%)	3 (3%)	0 (0%)	
*K. pneumoniae*	7 (4%)	4 (3%)	3 (7%)	
*S. aureus*	1 (1%)	1 (1%)	0 (0%)	
*E. faecalis*	8 (5%)	7 (6%)	1 (2%)	
*P. aeruginosa*	6 (4%)	2 (2%)	4 (9%)	
*P. mirabilis*	3 (2%)	2 (2%)	1 (2%)	
*Citrobacter*	3 (2%)	3 (3%)	0 (0%)	
*E. faecium*	1 (1%)	1 (1%)	0 (0%)	
Others *	5 (3%)	2 (2%)	3 (7%)	
Second Germ in Urine Culture	34 (23%)	29 (28%)	5 (11%)	0.023
Blood Culture Positive	19 (12%)	7 (6%)	12 (26%)	<0.001
Same Germ in blood and urine	10 (6%)	2 (2%)	8 (17%)	<0.001
CRP	46 (14–95)	38 (10–95)	55 (28–99)	0.17
PCT	1 (0–3)	0 (0–1)	2 (1–6)	0.002

**Table 2:** Demographic and microbiological data of patients according to blood culture positivity. Data are presented as a mean (SD) or median (IQR) for continuous measures as appropriate and n (%) for categorical measures. N: number.

	Total	Negative Blood Culture	Positive Blood Culture	*p*-Value
	N = 161	N = 142	N = 19	
Age (months)	3.0 (1.0–8.0)	3.0 (1.0–8.0)	2.0 (1.0–36.0)	0.55
Female Sex	68 (42%)	64 (45%)	4 (21%)	0.047
Bacteria found in Urine culture				0.70
*E. coli*	124 (77%)	105 (74%)	19 (100%)	
*K. oxytoca*	3 (2%)	3 (2%)	0 (0%)	
*K. pneumiae*	7 (4%)	7 (5%)	0 (0%)	
*S. aureus*	1 (1%)	1 (1%)	0 (0%)	
*E. faecalis*	8 (5%)	8 (6%)	0 (0%)	
*P aeruginosa*	6 (4%)	6 (4%)	0 (0%)	
*P Mirabilis*	3 (2%)	3 (2%)	0 (0%)	
Citrobacter	3 (2%)	3 (2%)	0 (0%)	
*E faecium*	1 (1%)	1 (1%)	0 (0%)	
Others *	5 (3%)	5 (4%)	0 (0%)	
Second Bacteria in Urine culture				0.95
No second bacteria	127 (79%)	110 (77%)	17 (89%)	
*E. coli*	3 (2%)	3 (2%)	0 (0%)	
*K. oxytoca*	1 (1%)	1 (1%)	0 (0%)	
*E. fecalis*	17 (11%)	16 (11%)	1 (5%)	
*P. aeruginosa*	1 (1%)	1 (1%)	0 (0%)	
*P. mirabilis*	1 (1%)	1 (1%)	0 (0%)	
*Citrobacter*	1 (1%)	1 (1%)	0 (0%)	
*E. fecium*	2 (1%)	2 (1%)	0 (0%)	
*S. faecalis*	4 (2%)	3 (2%)	1 (5%)	
Others *	4 (2%)	4 (3%)	0 (0%)	
Same Germ in blood and urine	10 (6%)	0 (0%)	10 (53%)	<0.001
Urinary tract malformation	46 (29%)	34 (24%)	12 (63%)	<0.001
CRP	46 (14–95)	40 (12–95)	55 (42–126)	0.13
PCT	1 (0–3)	0 (0–2)	3 (1–12)	<0.001

* means other bacteria.

**Table 3:** Multivariate analysis for the outcome of “presence of urinary abnormalities”.

Presence of Urinary Abnormalities	Odds Ratio	Std. Err.	z	*p * > z	[95% Conf. Interval]
**Urine culture positive for *P. aeroginosa* or unusual germs**	8.15	5.46	3.13	0.002	2.18–30.36
**Age**	1.01	0.003	3.33	0.001	1.005–1.020
**Blood Culture positivity**	6.5	3.55	3.44	0.001	2.23–18.98

**Table 4:** Predicted probability of a urinary anomaly according to the bacteria encountered in the urine culture while holding other covariants as constant.

Bacteria in Urineculture	Predicted Probability	Std. Err.	z	*p* > z	[95% Conf. Interval]
***E. coli***	0.27	0.04	6.84	<0.001	0.19–0.35
***K. pneumoniae***	0.43	0.18	2.29	0.022	0.06–0.79
***E. faecalis***	0.12	0.12	1.07	0.285	−0.10–0.35
***P. aeruginosa***	0.67	0.19	3.46	0.001	0.28–1.04
***P. mirabilis***	0.33	0.27	1.22	0.221	−0.20–0.86
**Unusual germs**	0.6	0.21	2.74	0.006	0.17–1.02

## Data Availability

The datasets generated during and/or analysed during the current study are available from the corresponding author on reasonable request. The data are not publicly available due to local rules.
